# Tumor-intrinsic signaling pathways: key roles in the regulation of the immunosuppressive tumor microenvironment

**DOI:** 10.1186/s13045-019-0804-8

**Published:** 2019-11-27

**Authors:** Li Yang, Aitian Li, Qingyang Lei, Yi Zhang

**Affiliations:** 1 Biotherapy Center, The First Affiliated Hospital of Zhengzhou University, Zhengzhou, Henan 450052 People’s Republic of China; 2 Cancer Center, The First Affiliated Hospital of Zhengzhou University, Zhengzhou, Henan 450052 People’s Republic of China; 3 School of Life Sciences, Zhengzhou University, Zhengzhou, Henan 450001 People’s Republic of China; 4 Henan Key Laboratory for Tumor Immunology and Biotherapy, Zhengzhou, Henan 450052 People’s Republic of China

**Keywords:** Immunosuppressive tumor microenvironment, Immune escape, T cell infiltration, Immunosuppressive cells, Tumor-intrinsic signaling

## Abstract

Immunotherapy is a currently popular treatment strategy for cancer patients. Although recent developments in cancer immunotherapy have had significant clinical impact, only a subset of patients exhibits clinical response. Therefore, understanding the molecular mechanisms of immunotherapy resistance is necessary. The mechanisms of immune escape appear to consist of two distinct tumor characteristics: a decrease in effective immunocyte infiltration and function and the accumulation of immunosuppressive cells in the tumor microenvironment. Several host-derived factors may also contribute to immune escape. Moreover, inter-patient heterogeneity predominantly results from differences in somatic mutations between cancers, which has led to the hypothesis that differential activation of specific tumor-intrinsic pathways may explain the phenomenon of immune exclusion in a subset of cancers. Increasing evidence has also shown that tumor-intrinsic signaling plays a key role in regulating the immunosuppressive tumor microenvironment and tumor immune escape. Therefore, understanding the mechanisms underlying immune avoidance mediated by tumor-intrinsic signaling may help identify new therapeutic targets for expanding the efficacy of cancer immunotherapies.

## Background

The recent developments in cancer immunotherapy show significant clinical impact. Particularly, monoclonal antibodies targeting the immune checkpoints cytotoxic T-lymphocyte-associated protein 4 (CTLA-4) and programmed cell death protein 1 (PD-1) have shown dramatic efficacy and have been approved by the FDA for cancer treatment [1–4]. Nevertheless, only a subset of patients experiences clinical benefit. Furthermore, chimeric antigen receptor-T (CAR-T) cell therapy has been approved for the treatment of certain hematological malignancies, yet solid cancers are often less susceptible to CAR-T cell therapy mostly due to the immunosuppressive tumor microenvironment [5, 6]. Therefore, understanding the molecular mechanisms of immunotherapy resistance, specifically those induced by the tumor microenvironment, is necessary.

The tumor microenvironment consists of the non-cancerous cells present in the tumor, which includes immune cells, fibroblasts, and cells that comprise the blood vessels [7, 8]. It has been shown that a subset of melanoma patients with metastases exhibits a T cell-inflamed tumor microenvironment as evidenced by gene expression profiling [9]. The T cell-inflamed phenotype also shows activated immune-inhibitory pathways as well as expression of PD-L1 and indoleamine-2,3-dioxygenase (IDO) [10]. In contrast, the lack of T cell infiltration in the tumor microenvironment appears to avoid antitumor immunity through the exclusion of T cells from the tumor site. In addition, immunosuppressive cells, including tumor-associated macrophages (TAMs), myeloid-derived suppressor cells (MDSCs), T regulatory cells (Tregs), and tumor-associated neutrophils (TANs), are also responsible for an immunosuppressive tumor microenvironment and tumor immune escape [7, 11–13]. Thus, the mechanisms of immune escape appear to be distinct in two major subsets of tumors, that is, a decrease in effective immunocyte infiltration and function and an increase in immunosuppressive cells in the tumor microenvironment.

Several host-derived factors may also contribute to immune escape. Inter-patient heterogeneity predominantly results from differences in somatic mutations between cancers [14], which has led to the hypothesis that differential activation of specific tumor-intrinsic pathways may explain the phenomenon of immune exclusion in a subset of cancers. In addition to the activation of tumor-intrinsic pathways within the tumor cells themselves, exposure to chronic viral infections, the composition of the intestinal microbiota of patients, and the accumulation of germline polymorphisms in immune regulatory genes may also influence the antitumor immunotherapy response [15, 16].

Tumor-intrinsic signaling pathways are considered to be oncogenic pathways. Increasing evidence has shown that tumor-intrinsic signaling plays a key role in regulating the immunosuppressive tumor microenvironment and tumor immune escape [17, 18]. Successful identification of these pathways would lead to new therapeutic strategies that can enable immunocyte entry into non-inflamed tumors and attenuate the immunosuppressive microenvironment to increase the number of patients capable of responding to immunotherapies. In this review, we will describe the mechanisms by which tumor-intrinsic signaling pathways regulate the immunosuppressive tumor microenvironment, including the decrease in effective immunocyte infiltration and function and the accumulation of immunosuppressive cells in the tumor microenvironment, which may help identify new therapeutic targets for enhancing the efficacy of cancer immunotherapy.

## Effective immunocyte exclusion and dysfunction

**Fig. 1:**
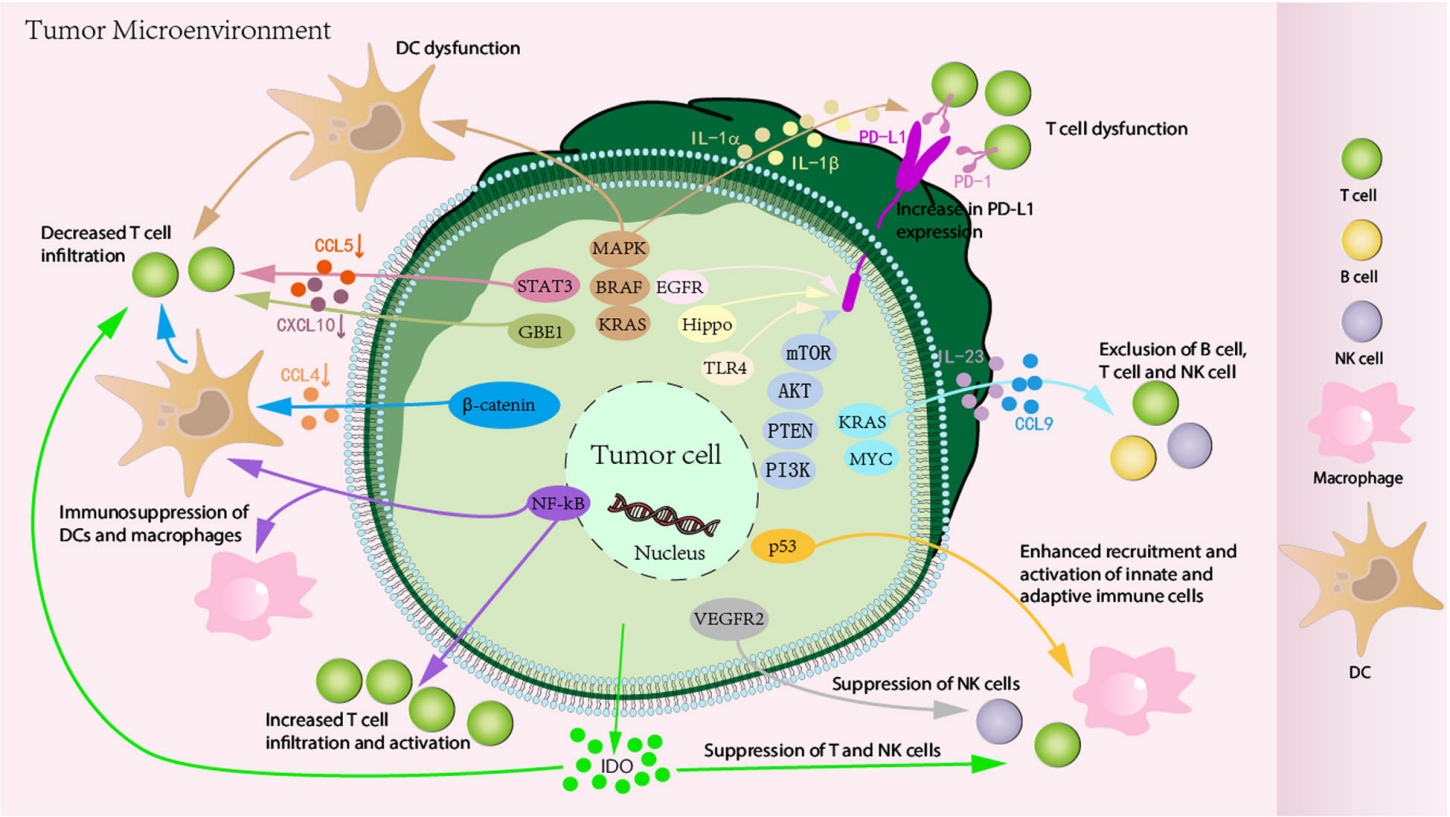
Tumor-intrinsic signaling induces the exclusion and dysfunction of effective immunocytes. Oncogenic drivers of tumors, including β-catenin, STAT3, PI3K/PTEN/AKT/mTOR, p53, NF-κB, and RAS/RAF/MAPK signaling, are activated in the tumor microenvironment. These oncogenic signaling pathways not only downregulate the production of chemokines, which further decrease the recruitment of DCs, macrophages, T cells, and NK cells to tumor sites, but also induce immunosuppression of these immunocytes. In addition, tumor-intrinsic signaling can induce PD-L1 expression in tumor cells, leading to T cell dysfunction in the tumor microenvironment

The innate and adaptive immune cells in the tumor microenvironment harbor both tumor-promoting and tumor-suppressing activities, which may predict clinical outcome [19, 20]. It has been shown that oncogenic drivers of tumors may function to limit host immunity in the remaining non-immunocyte inflamed tumors or dysfunction of immunocytes in the tumor microenvironment, thereby leading to immunoresistance (Fig. 1, Table 1).

**Table 1:** The influence of different tumor-intrinsic signaling pathways in different cancers

Subtype	Signaling	Tumor type	Effect	Ref
Effective immunocyte exclusion and dysfunction	β-Catenin	Melanoma	Decreased T cell infiltration	18, 21, 22
			Inhibition of IFN-γ production by CTLs	23
			Upregulating the expression and activity of IDO by DCs	24
	STAT3	Lung cancer	Inhibition of CCL5 and CXCL10 production to decrease T cell infiltration	25, 26, 28
	PI3K/PTEN/AKT/mTOR	Breast, prostate, and lung cancer, gliomas	Regulation of PD-L1 expression to induce T cell dysfunction	29, 31–33
		Triple-negative breast cancer	Decreased T cell infiltration, regulation of PD-L1 expression	30
		Multiple cancers	Decreased the therapeutic efficacy of an E7-specific vaccine or CD8^+^ T cell adoptive transfer	34
	p53	Liver carcinoma	Increased recruitment and activation of innate immune cells	37,38
		Triple-negative breast cancer	Regulation of T cell infiltration	39
	NF-κB	Epithelial ovarian cancer	Immunosuppression of DCs and macrophages	42
		Colitis-associated cancer, cervical cancer, etc.	Increased T cell infiltration and activation	43–46
	RAS/RAF/MAPK	Lung adenocarcinoma, RAS mutant cancer	Inducing PD-L1 expression	47, 48
		Melanoma	Suppression of DC function	50, 51
		Melanoma	Inhibiting the recognition of tumor cell antigens by tumor-infiltrated T lymphocytes	52
		Melanoma	Suppression of proliferation and function of specific cytotoxic T cells	53
	GBE1	Lung adenocarcinoma	Decreased T cell infiltration	54
	KRAS/MYC	KRAS-mutant tumor	Exclusion of B, T, and NK cells	55
	EGFR	Non-small cell lung cancer, head and neck cancer	Upregulation of PD-L1 expression	56–60
	VEGFR	Chronic myeloid leukemia	Inhibited NK cell-mediated immunosurveillance	61
Recruitment and differentiation of immunosuppressive cells	PI3K/PTEN/AKT	Breast, pancreatic, and lung carcinomas	Recruitment of macrophages and polarization of TAMs	70–72
		Sarcomas	Enhanced infiltrating myeloid-derived hematopoietic cells	73
		Prostate cancer	Increased expansion and infiltration of MDSCs	74,75
	RAS/RAF/MAPK	KRAS-driven lung tumorigenesis, melanoma	Increased Treg infiltration	76,78
		BRAFi-resistant melanoma	Increased MDSC infiltration	77
	KRAS	KRAS-driven non-small cell lung cancer	Accumulation of TANs	79
		KRAS-mutant tumor	Recruitment of proangiogenic macrophages	55
	CCRK/mTOR	Obesity-associated hepatocellular carcinoma	Recruitment of MDSCs	80
	RAGE	Pancreatic carcinogenesis	Accumulation of MDSCs	81
	TLR9	Prostate cancer	Expansion and activation of G-MDSCs	82
	p53 loss-of-function	Late stage metastatic castration resistant prostate cancer	Accumulation of MDSCs	83
	IDO	Advanced cancer	Generation and activation of MDSCs and Tregs	64
	CD200/CD200R	Chemical skin carcinogenesis	Influencing the ratio of Treg/Th17 cells	84, 85
	STAT3	Hematopoietic system	Recruiting and promoting the proliferation of Tregs	86, 87
	COX2	Wilms' tumor	Increased Treg infiltration	90
	c-MET	Melanoma	Increased TAN infiltration	91

### β-Catenin signaling

Differential activation of the β-catenin oncogene pathway within tumor cells themselves contributes to the robustness of a spontaneous antitumor immune response (Fig. 1, Table 1). Recently, Spranger et al. [21] found that 48% of the non-T cell-inflamed tumors show evidence of WNT/β-catenin signaling pathway activation based on gene expression profiling of six defined β-catenin target genes. In vivo experiments demonstrated that activation of the β-catenin pathway within melanoma tumor cells can dominantly exclude immune cell activation and result in a non-T cell-inflamed tumor microenvironment. β-Catenin-mediated immune escape occurs via inhibition of the production of CCL4 derived from tumor cells; this results from induction of the transcriptional repressor ATF3, which blocks CCL4 gene transcription. The lack of CCL4 secretion results in decreased recruitment of CD103^+^ dendritic cells (DCs), thereby preventing cross-priming of antitumor T cells [18, 22]. In addition, β-catenin-overexpressed melanomas inhibit the production of IFN-γ by melanoma-specific cytotoxic lymphocytes (CTLs) in an interleukin (IL)-10-independent manner and were more resistant to CTL lysis in vitro and in vivo [23]. Moreover, melanoma-derived Wnt5a ligand upregulates the durable expression and activity of IDO enzyme by local DCs in a β-catenin signaling pathway-dependent manner [24].

### STAT3 signaling

One potential candidate for oncogenic drivers leading to immunoresistance is activation of the STAT3 signaling pathway (Fig. 1, Table 1). Constitutively active STAT3 signaling in transplantable tumor cell lines has been reported to decrease expression of proinflammatory mediators, while expression of a dominant negative STAT3 variant resulted in augmented expression of proinflammatory factors, including the chemokines CCL5 and CXCL10, which are functionally responsible for T cell recruitment [25, 26]. Recent studies have provided additional evidence for this phenomenon via a carcinogen-induced lung cancer model and a genetically-induced prostate cancer model [27, 28]. Using a conditional knockout model for STAT3, Ihara et al. [28] found an increased antitumor immune response in the absence of STAT3 signaling, which was closely associated with increased expression of CCL5 and CXCL10; this phenotype was associated with increased T cell infiltration and function within the tumor microenvironment. Thus, the STAT3 signaling pathway may represent a viable mechanistic pathway for diminishing immune cell recruitment into tumor sites, and based on the currently available data, it may interfere with T cell recruitment.

### PI3K/PTEN/AKT/mTOR signaling

The PI3K/PTEN/AKT/mTOR pathway is another interesting candidate that may impact the host immune response (Fig. 1, Table 1). The expression of PD-L1, a pivotal negative regulator of T cell function, is associated with the activation of PI3K in breast and prostate cancer patients [29]. Recent findings have demonstrated that the expression of tumor suppressor PTEN was closely associated with the lack of T cell infiltration as well as low PD-L1 expression in the tumor microenvironment of triple-negative breast cancer [30], indicating that loss of PTEN expression (and constitutive PI3K activation) is associated with the presence of T cells in the tumor microenvironment. In the LKB1, PTEN-null model, tumor-propagating cells of human lung squamous cell carcinoma highly expressed PD-L1, suggesting a mechanism of immune escape for tumor-propagating cells [31]. Moreover, loss of PTEN function increases PD-L1 expression and immunoresistance in gliomas [32]. Furthermore, oncogenic activation of the AKT-mTOR signaling pathway promotes immune escape by driving the expression of PD-L1, which was confirmed in syngeneic and genetically engineered mouse models of lung cancer where combination therapy of an mTOR inhibitor with a PD-1 antibody decreased tumor growth and increased T cell infiltration [33]. Intratumoral injection of an AKT inhibitor also enhanced the therapeutic efficacy of an E7-specific vaccine or E7-specific CD8^+^ T cell adoptive transfer against immune-resistant tumors [34]. These findings indicate that activation of the PI3K/AKT signaling pathway represents a new mechanism of immune escape that has important implications for the development of a novel cancer immunotherapy strategy against immune-resistant tumors.

### p53 signaling

Mutant p53 is another molecular aberration in cancer cells that is associated with immune response (Fig. 1, Table 1). Activating/reactivating p53 signaling in the tumor microenvironment represents a compelling immunological strategy for enhancing antitumor immunity and reversing immunosuppression [35, 36]. It has been shown that an intact p53 signaling pathway is correlated with increased recruitment and activation of innate immune cells [37]. In a related study, where cellular senescence is triggered in vivo by inducible p53 expression using a mouse model of liver carcinoma, tumor regression associated with re-expression of wildtype p53 was strongly dependent on the activation and recruitment of natural killer (NK) cells into the tumor site [38]. Consistent with these findings, a recent study tested for interaction between TP53 mutation status and integrative cluster analysis in 1420 breast tumors, indicating a close correlation between wildtype p53 and the presence of T cells in the tumor microenvironment of triple-negative breast cancer [39]. Moreover, in a murine liver carcinoma model, reactivation of p53 signaling induced tumor regression, which was associated with increased expression of proinflammatory chemokines. Collectively, these findings suggest that steady-state p53 signaling can contribute to enhanced recruitment of innate and adaptive immune cells as well as their activation.

### NF-κB signaling

Another candidate oncogenic signaling pathway that has potential effects on the host immune response is the NF-κB signaling pathway (Fig. 1, Table 1). Activation of this pathway in cancer cells has been associated with tumor progression [40, 41]. In epithelial ovarian cancer patients, increased plasma IL-6, IL-8, and arginase were observed, and the NF-κB inhibitor DHMEQ inhibited the production of IL-6 and IL-8 by epithelial ovarian cancer cell lines. Treatment with DHMEQ reversed the immunosuppression of human DCs and macrophages cultured in the supernatant of epithelial ovarian cancer cells [42]. The NF-κB signaling pathway induces the production of cytokines that regulate the immune response (e.g., TNFα, IL-1, IL-6, and IL-8) as well as adhesion molecules that lead to the recruitment of leukocytes into tumor sites [43]. Constitutive activation of NF-κB has been shown to increase the expression of tumor cell-derived chemokines, which can have positive immune effects [44]. Activation of NF-κB signaling also increases the production of chemokines that can recruit activated T cells within the tumor microenvironment [45]. Moreover, full activation of NF-κB is accompanied by increased activity of cytotoxic immune cells against cancer cells in early cancer stages [46]. Therefore, the impact of tumor-intrinsic NF-κB signaling activation on host immunity may depend on the cellular context.

### RAS/RAF/MAPK signaling

The RAS/RAF/MAPK pathway is probably the best characterized signal transduction pathway in cell biology. The function of this pathway is to transduce signals from the extracellular milieu to the cell nucleus where specific genes are activated for cell growth, differentiation, and migration. Thus, the RAS/RAF/MAPK signaling regulates a variety of cellular functions that are important for tumorigenesis.

The RAS/RAF/MAPK signaling pathway is also involved in the host immune response (Fig. 1, Table 1). KRAS mutations induce PD-L1 expression through p-ERK signaling in lung adenocarcinomas. Blockade of PD-1/PD-L1 signaling would thus be a promising therapeutic strategy for KRAS-mutant lung adenocarcinoma [47]. Similarly, Coelho et al. [48] found that oncogenic KRAS signaling increases PD-L1 expression in tumor cells.

Because DCs are important in the induction of tumor-specific T cell responses, the effect of MAPK pathway activation on DC function is critical for the melanoma-directed immune response [49]. BRAF^V600E^ mutant melanoma cells regulate DCs through the MAPK signaling pathway, whose blockade can reverse the suppression of DC function. The inhibition of MEK, a MAPK/ERK kinase, negatively impacts DC function and viability [50]. The suppressive activity of melanoma cell culture supernatants on the production of IL-12 and TNFα by DCs upon lipopolysaccharide stimulation was significantly reduced after transduction with BRAF^V600E^ RNAi [51]. In addition, blocking the BRAF-MAPK signaling pathway in BRAF signaling-addicted melanoma cells in vitro triggered the recognition of tumor cell antigens by tumor-infiltrated T lymphocytes; BRAF blockade and adoptive T cell therapy may confer synergistic effects [52]. Moreover, the expression of BRAF^V600E^ induced transcription of IL-1α and IL-1β in melanocytes and melanoma cell lines, which increased the suppression of proliferation and function of specific cytotoxic T cells in melanomas [53].

### Other signaling

Other oncogenic signaling pathways also contribute to tumor immune escape (Fig. 1, Table 1). In our previous study, lung adenocarcinoma-intrinsic glycogen branching enzyme (GBE1) signaling was found to inhibit antitumor immunity. GBE1 blockade promotes the secretion of CCL5 and CXCL10 to recruit CD8^+^ T lymphocytes to the tumor microenvironment via the IFN-I/STING signaling pathway, accompanied by upregulation of PD-L1 in lung adenocarcinoma cells; this indicates that GBE1 is a promising cancer immunotherapy target for achieving tumor regression in lung adenocarcinomas [54].

Immune suppression in KRAS-mutant mouse tumors with co-activation of MYC may lead to increased expression of IL-23 and CCL9, which mediate the exclusion of B, T, and NK cells [55].

EGFR is also involved in the regulation of PD-L1 expression in non-small cell lung cancer [56–58], which suppresses T cell function. Overexpression of EGFR is correlated with PD-L1 expression in head and neck cancers in a JAK2/STAT1-dependent manner, indicating a novel role for JAK2/STAT1 in EGFR-induced immune evasion [59]. This study found that PD-L1 expression increased significantly in an EGFR-dependent manner by the activation of EGFR signaling and decreased sharply when EGFR signaling was blocked. The upregulated expression of PD-L1 was not associated with EGFR/STAT3 signaling pathway, but may be affected by EGFR/PI3K/AKT, EGFR/RAS/RAF/ERK, and EGR/PLC-γ signaling pathways [60]. VEGFR2-targeted fusion antibody improved NK cell-mediated immunosurveillance against K562 cells through increasing degranulation and cytokine production of NK cells [61].

Human-specific activation of PD-L1 by a novel Hippo signaling pathway in cancer immune evasion may have a significant impact on immunotherapy research [62]. Moreover, inactivation of Hippo signaling in tumor cells induces a type I interferon response, increases tumor immunogenicity, and enhances tumor vaccine efficacy [63].

Meanwhile, IDO activation in cancers mediates the suppression of T and NK cells [64, 65]. Hennequart et al. [66] highlighted the role of COX-2 in constitutive IDO1 expression by human tumors and demonstrated that COX-2 inhibitors can reduce constitutive IDO1 expression, which contributes to the lack of T cell infiltration in “cold” tumors that fail to respond to immunotherapy. Moretti et al. [67] provided the first evidence of a direct link between IDO1 expression and oncogenic activation of RET in thyroid carcinoma and described the involved signal transduction pathways.

Finally, activation of TLR4 signaling in bladder cancer cells upregulates PD-L1 expression [68]. Isocitrate dehydrogenase mutations in glioma cells lead to acquired resistance to NK cells through epigenetic silencing of NKG2D ligands [69].

## Recruitment and differentiation of immunosuppressive cells

**Fig. 2:**
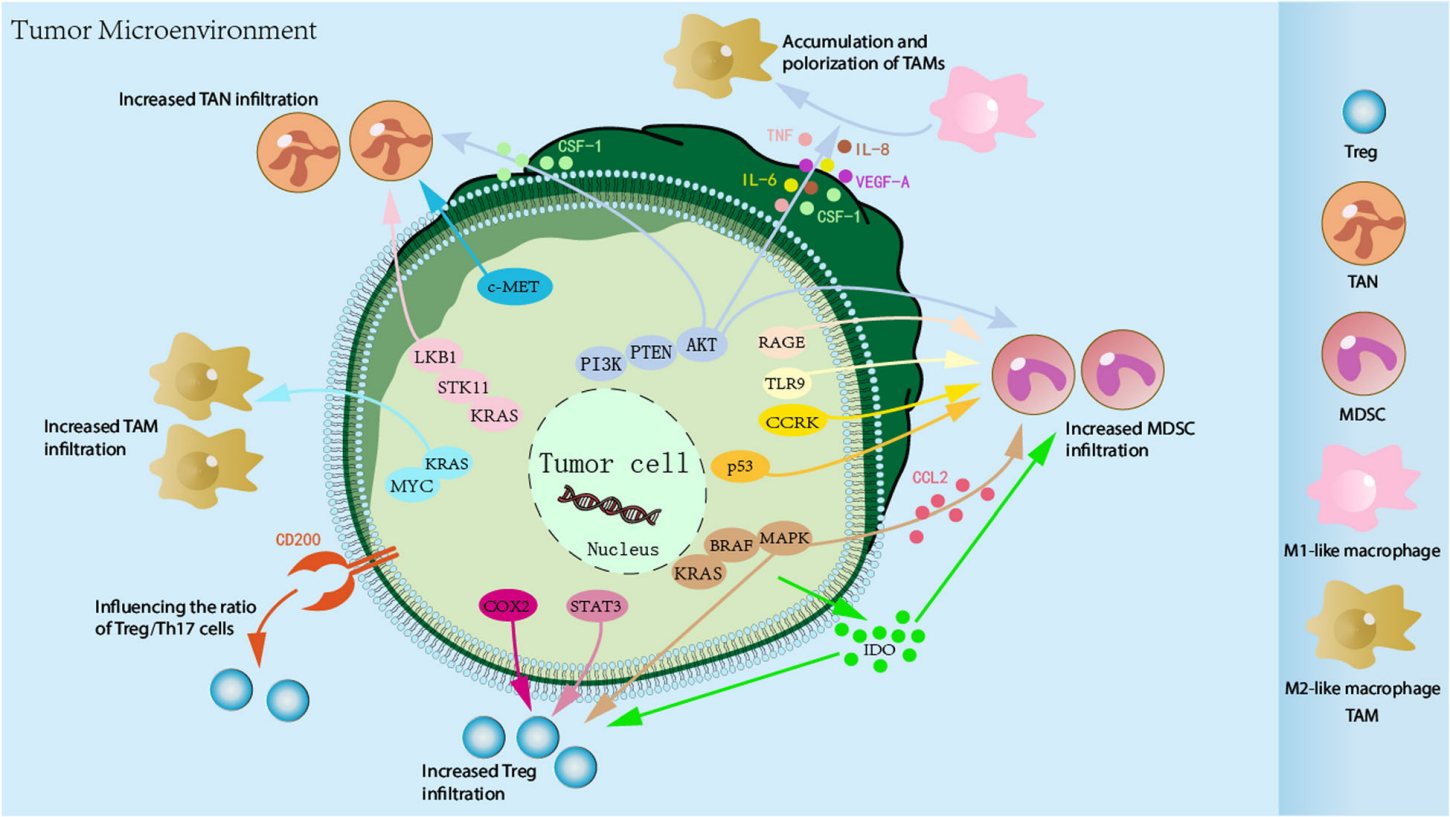
Tumor-intrinsic signaling mediates the recruitment and differentiation of immunosuppressive cells. Oncogenic pathways in tumor cells can be activated to promote the production of several chemokines and cytokines, which further enhance the recruitment and polarization of immunosuppressive cells, such as TAMs, MDSCs, Tregs, and TANs, to tumor sites. These immunosuppressive cells within the tumor microenvironment may also contribute to immunoresistance in cancers

In addition to alterations in T cell immune checkpoints, an increase in immunosuppressive cells, including TAMs, MDSCs, Tregs, and TANs, and differentiation of these immunosuppressive cells within the tumor microenvironment may also contribute to immunoresistance in cancers (Fig. 2, Table 1).

### PI3K/PTEN/AKT signaling

The PI3K/PTEN/AKT oncogenic signaling pathway has a positive effect on immunosuppressive cell recruitment and differentiation (Fig. 2, Table 1). Several studies have demonstrated that activated PI3K signaling, either through activating mutations in PIK3CA or loss-of-function mutations in PTEN, can result in the accumulation of TAMs, which induce an immunosuppressive microenvironment [70, 71]. This phenomenon was associated with increased production of TNF, IL-6, CSF-1, VEGF-A, and IL-8 by tumor cells, which contribute to the recruitment of macrophages and the polarization of M2-like macrophages [72]. PTEN-deficient sarcomas exhibit enhanced infiltrating myeloid-derived hematopoietic cells, particularly macrophages and neutrophils, recruited via tumor cell-derived CSF-1 [73]. Furthermore, PTEN-null prostate epithelium triggers the production of inflammatory cytokines and mediates localized Gr-1^+^CD11b^+^ MDSC expansion and immune suppression, thereby promoting tumor progression [74]. In genetically engineered mouse models of prostate cancer, the deletion of PTEN and Smad4 promotes tumor progression and infiltration of MDSCs [75].

### RAS/RAF/MAPK signaling

The RAS/RAF/MAPK signaling pathway is also involved in the recruitment and differentiation of immunosuppressive cells (Fig. 2, Table 1). Overexpression of the mutant KRAS G12V gene in wildtype KRAS tumor cells led to Treg induction through the activation of the MEK-ERK-AP1 pathway, while KRAS inhibition reduced Treg infiltration in KRAS-driven lung tumorigenesis even before tumor formation [76].

Preclinical studies showed that treatment with BRAF^V600E^ inhibitors (BRAFi) initially reduced MDSC infiltration in the tumor microenvironment of an autochthonous mouse model of melanoma, but resistance to BRAFi was associated with restoration of MDSCs. In contrast to the restoration of MDSCs, Treg levels remained low in BRAFi-resistant tumors. Notably, MDSC restoration relied upon the reactivation of MAPK signaling and downstream production of CCL2, the myeloid attractant, in BRAFi-resistant melanoma cells [77]. Shabaneh et al. [78] found that BRAF^V600E^ signaling was sufficient to recruit Tregs into the tumor microenvironment, establishing a novel role for BRAF^V600E^ as a tumor-intrinsic mediator of immune escape and underscoring the critical early role of Treg-mediated suppression during tumorigenesis.

### KRAS signaling

The KRAS signaling pathway cooperated with other molecules is also involved in the recruitment and differentiation of immunosuppressive cells (Fig. 2, Table 1). In a mouse model of KRAS-driven non-small cell lung cancer, STK11/LKB1 loss was found to affect the immune microenvironment. Genetic ablation of STK11/LKB1 resulted in the accumulation of TANs, which results in T cell-suppressive effects along with a corresponding increase in the expression of T cell exhaustion markers and tumor-promoting cytokines [79]. In KRAS-mutant mouse tumors, immune suppression may be a result of MYC co-activation leading to the recruitment of proangiogenic macrophages in the tumor microenvironment [55].

### Other signaling

Other oncogenic events common in cancer, such as infiltration and differentiation of MDSCs, TANs, and Tregs, may also have the potential to enhance the immunosuppressive tumor microenvironment (Fig. 2, Table 1).

Hepatic cell cycle-related kinase (CCRK) induction in transgenic mice stimulates mTORC1-dependent G-CSF secretion, which further enhances the recruitment of polymorphonuclear MDSCs [80]. These findings indicate a role for an inflammatory-CCRK signaling pathway in driving immunosuppressive reprogramming through the activation of mTORC1, thereby reeducating the pro-tumorigenic microenvironment of hepatocellular carcinoma. The receptor for advanced glycation end-products (RAGE) promotes accumulation of MDSCs to further induce pancreatic carcinogenesis [81]. Moreover, TLR9^+^ prostate cancer promotes immune evasion via LIF-mediated expansion and activation of G-MDSCs [82]. In preclinical melanoma mouse models, p53 loss-of-function promotes the accumulation of MDSCs within the tumor microenvironment of late stage metastatic castration resistant prostate cancer [83]. The activation of IDO in cancers can also induce the generation and activation of MDSCs and Tregs [64].

In another study, the CD200/CD200R axis was shown to induce tolerance to external and tumor antigens and to influence the ratio of Treg/Th17 cells and control the balance of Treg/T effector cells, which provides a therapeutic strategy for CD200 blocking antibodies [84, 85]. The STAT3 signaling pathway also plays an important role in recruiting and promoting the proliferation of Tregs [86, 87], which in turn has suppressive activity toward CD8^+^ effector T cells and other immune cell types within the tumor microenvironment [88, 89]. Moreover, COX2 signaling can increase the infiltration of immune suppressive inflammatory cells, such as Tregs, in tumors [90].

Finally, a study by Glodde et al. [91] showed that c-MET inhibition impairs reactive TAN recruitment to tumors and lymph nodes, potentiating T cell antitumor immunity.

## Therapeutic targets for tumor-intrinsic signaling in cancer

**Fig. 3:**
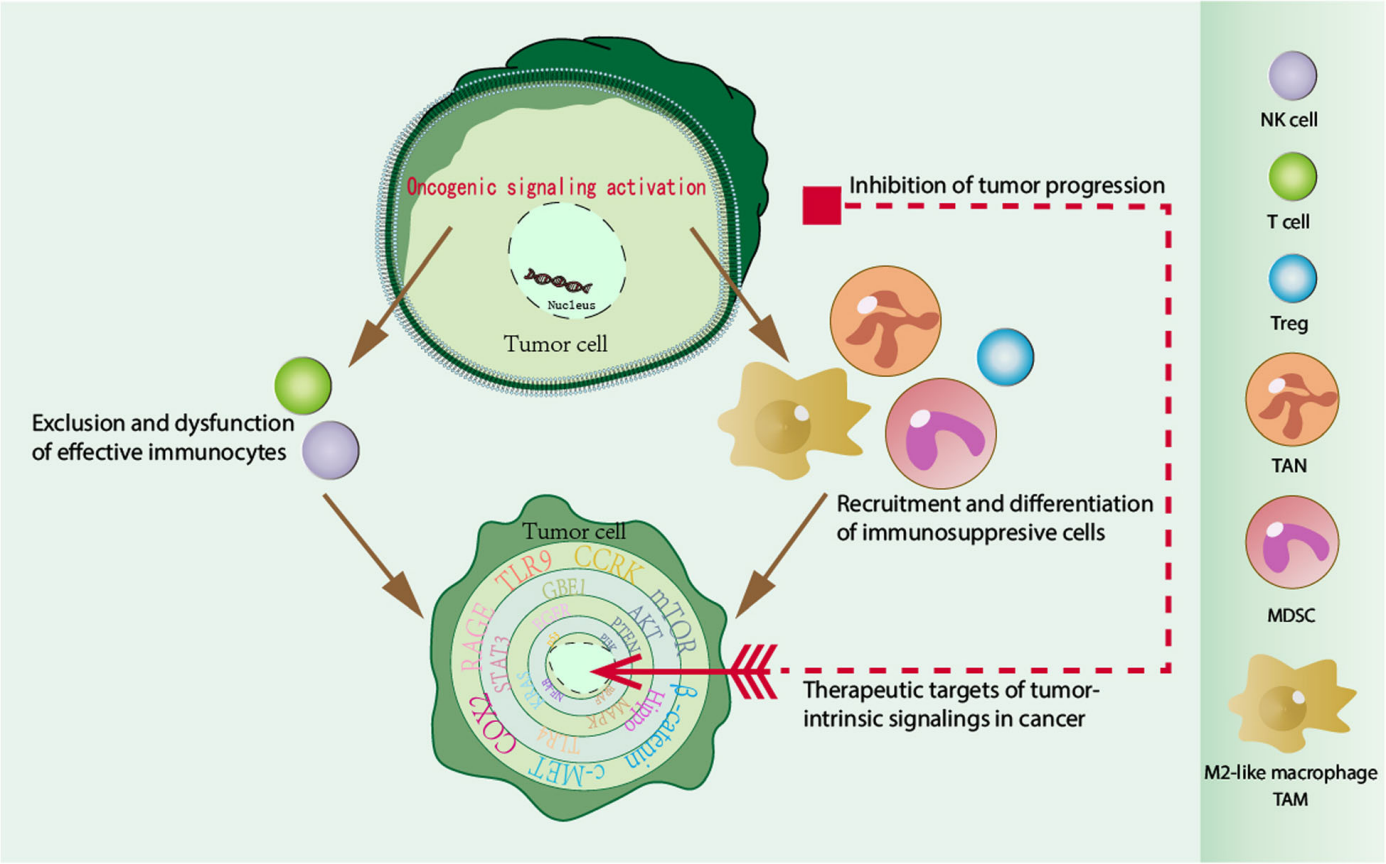
Tumor-intrinsic signaling as a therapeutic target for cancers. The activation of tumor-intrinsic signaling regulates and promotes the immunosuppressive tumor microenvironment, which includes exclusion and dysfunction of effective immunocytes and recruitment and differentiation of immunosuppressive cells. Therefore, targeting the tumor-intrinsic signaling is a potential strategy for cancer treatment

As discussed above, there is strong evidence that tumor-intrinsic signaling regulates the immunosuppressive tumor microenvironment via exclusion and dysfunction of effective immunocytes and recruitment and differentiation of immunosuppressive cells. Therefore, targeting tumor-intrinsic signaling is a promising strategy for cancer treatment. In the following sections, we will discuss therapeutic strategies for targeting oncogenic signaling (Table 2, Fig. 3).

**Table 2:** Therapeutic strategies of targeting tumor-intrinsic signaling in preclinical studies and clinical trials

Target	Therapeutic agent	Phase	Tumor type	Effect	Trial number	Ref
BRAF	Vemurafenib	III	BRAF(V600) mutation-positive melanoma	Well tolerated	NCT01667419	92
BRAF/MEK	Vemurafenib + cobimetinib	Ib	Advanced BRAF-mutated melanoma	Safe and tolerable	NCT01271803	93
	Vemurafenib + cobimetinib	III	Advanced BRAF^V600^-mutant melanoma	Improved progression-free survival, increased toxicity	NCT01689519	94, 95
	Dabrafenib + trametinib	III	BRAF^V600^-mutant metastatic melanoma	Durable (≥ 3 years) survival is achievable	NCT01584648	96
	Dabrafenib + trametinib	III	BRAF^V600^-mutant unresectable or metastatic melanoma	Survival advantage	NCT01597908	97
	Dabrafenib + trametinib	III	Metastatic melanoma with BRAF^V600^ mutation	Improved overall survival	NCT01597908	98
	Dabrafenib + trametinib	III	Melanoma with BRAF^V600^ mutation	Significantly lower risk of recurrence	NCT01682083	99
	Dabrafenib + trametinib	II	Untreated BRAF^V600^-mutant non-small cell lung cancer	Meaningful antitumor activity, manageable safety profile	NCT01336634	100, 101
	Dabrafenib + trametinib	II	BRAF-mutant melanoma	Longer progression-free survival and duration of response with a higher rate of grade 3/4 adverse events	NCT02130466	102
MEK	Trametinib	II	Oral cavity squamous cell carcinoma	Clinical tumor responses	NCT01553851	103
IDO	Epacadostat	I	Advanced Solid Malignancies	Well tolerated, effectively normalized kynurenine levels	NCT01195311	105
	Epacadostat	II	Advanced epithelial ovarian, primary peritoneal, or fallopian tube cancer	Well tolerated, no significant efficacy in ovarian cancer	NCT01685255	106
	Indoximod	I	Advanced solid tumors	Safe, best response was stable disease for > 6 months in 5 patients	NCT00567931	107
	Navoximod	Ia	Recurrent advanced solid tumors	Well tolerated, decreased kynurenine levels in plasma	NCT02048709	108
	Indoximod + docetaxel	I	Metastatic solid tumors	Well tolerated, no increase in toxicities or pharmacokinetic interactions	NCI #HHSN261201100100C	110
	Indoximod + checkpoint inhibitors	II	Advanced melanoma	52% overall response rate	NA	109
	Navoximod + atezolizumab	I	Advanced cancers	Acceptable safety and tolerability	NCT02471846	111
CTNNB1 (β-catenin)	NTRC 0066-0	Xenograft model	CTNNB1 mutant cancers	Complete inhibition of tumor growth	NA	112
STAT3	Stattic + metformin	In vitro experiment	Brain cancer	Inhibited tumor initiating cells	NA	115
	Stattic + recombinant vaccinia virus VG9	Xenograft model	Solid tumors	Superior antitumor ability	NA	116
PI3K	Duvelisib	I	Relapsed/refractory T cell lymphoma	Promising clinical activity and an acceptable safety profile	NCT01476657	117, 118
PI3K/mTOR	Dactolisib	In vitro and in vivo experiments	Glioblastomas	Antitumor activity	NA	119
	Omipalisib	In vitro experiment	Oncogenically transformed cells from neurocutaneous melanocytosis	Inhibited clonogenic growth	NA	120
Akt	Akti-1/2	In vitro experiment	Breast cancer	An anticancer therapeutic strategy	NA	121
NF-κB	QNZ	In vitro and in vivo experiment	Colorectal cancer	Decreased cell invasion and migration abilities as well as expression of metastasis-related markers	NA	122
	PDTC	In vitro and in vivo experiments	Multidrug-resistant breast cancer	Tumor growth inhibition	NA	123
	SN50	In vitro and in vivo experiments	Malignant brain tumor	Loss of oncogenesis, differentiation of stem-like cells	NA	124
TLR4	Rapamycin	In vitro experiment	Colon cancer	Inhibited IL-6, PGE(2) production, and cell invasion	NA	125

### BRAF/MEK inhibitors

The BRIM8 study (NCT01667419) evaluated the effects of BRAF inhibitor vemurafenib monotherapy in patients with resected, BRAF^V600^-mutant melanomas and found that 1 year of vemurafenib was well tolerated but may not be an optimal treatment regimen [92]. The safety and efficacy of combined vemurafenib and MEK inhibitor cobimetinib in patients with advanced BRAF-mutated melanoma were also assessed; when administered at their respective maximum tolerated doses, vemurafenib and cobimetinib co-therapy was safe and well tolerated (NCT01271803). This combination therapy shows promising antitumor activity, and confirmatory clinical testing is ongoing [93]. Moreover, Ascierto et al. [94] reported on the clinical benefit of vemurafenib and cobimetinib combination therapy and supported its use as a standard first-line strategy for improving survival in patients with advanced BRAF^V600^-mutant melanoma. In addition, the combination therapy of vemurafenib and cobimetinib was closely associated with a significant improvement in progression-free survival among patients with BRAF^V600^-mutated metastatic melanoma, at the cost of some increase in toxicity (NCT01689519) [95].

Dabrafenib is another selective inhibitor of mutated forms of BRAF kinase, and trametinib is another inhibitor of MEK 1/2. It has been shown that prolonged survival of more than 3 years is achievable with dabrafenib plus trametinib in patients with BRAF^V600^-mutant metastatic melanoma, supporting the long-term first-line use of this combination therapy [96]. BRAF^V600^-mutant unresectable or metastatic melanoma patients treated with a combination of dabrafenib plus trametinib show a clear benefit over patients receiving vemurafenib monotherapy, such as survival advantage as well as avoidance of disease-associated and adverse-event-associated symptoms, which supports this combination therapy as a standard of care for this population [97]. Another study (NCT01597908) showed that dabrafenib plus trametinib significantly improved overall survival without increased overall toxicity in previously untreated patients with metastatic BRAF^V600^-mutation melanoma compared with that of vemurafenib monotherapy [98]. Adjuvant use of a dabrafenib plus trametinib combination therapy resulted in a significantly lower risk of recurrence in patients with stage III BRAF^V600^-mutation melanoma and was not associated with new toxic effects (NCT01682083) [99]. Moreover, dabrafenib combined with trametinib represents a novel therapeutic strategy with meaningful antitumor activity, as evidenced by studies on patients with previously untreated BRAF^V600^-mutant non-small cell lung cancer [100, 101]. In a phase II trial (NCT02130466), combination therapy with dabrafenib, trametinib, and pembrolizumab conferred longer progression-free survival and duration of response with a higher rate of grade 3/4 adverse events compared with that of dabrafenib and trametinib doublet therapy [102].

Moreover, Uppaluri et al. [103] performed a clinical trial to determine the tumor response of oral cavity squamous cell carcinoma to treatment with the MEK inhibitor trametinib and found that trametinib caused a significant reduction of RAS/MEK/ERK signaling pathway activation and clinical tumor response.

### IDO inhibitors

Over the past decade, tryptophan catabolism has been considered a mechanism of innate and adaptive immune tolerance. Tryptophan catabolism is a central signaling pathway that maintains homeostasis by inhibiting the immunity that would result from uncontrolled immune responses. It is driven by the key enzymes IDO1 and tryptophan-2,3-dioxygenase 2 (TDO), which result in local depletion of tryptophan and accumulation of tryptophan catabolites, including kynurenine and its derivatives. This regulation of metabolism leads to a local immunosuppressive microenvironment resulting from several mechanisms whose respective roles remain incompletely understood.

Drugs targeting this signaling pathway and specifically IDO1 have already underwent clinical trials with the aim to revert immunosuppression induced by cancers [104]. Recently, several studies have demonstrated a favorable pharmacokinetic profile for first-generation and second-generation IDO1 inhibitors (INCB024360, NLG919). A set of mechanistically distinct compounds, including epacadostat, indoximod, and navoximod, were the first to be evaluated as IDO inhibitors in clinical trials. In a phase I study, epacadostat was well tolerated and effectively normalized kynurenine levels [105]. However, there was no significant difference in efficacy between epacadostat and tamoxifen for the treatment of advanced epithelial ovarian cancer in a phase II clinical trial [106]. Data from a phase I trial demonstrated that indoximod was safe at doses up to 2000 mg orally twice/day in patients with advanced solid tumors, and the best response was stable disease for > 6 months in five patients; however, induction of hypophysitis, increased tumor antigen autoantibodies, and C-reactive protein levels were observed [107]. A phase Ia study of navoximod (GDC-0919) treatment of patients with recurrent advanced solid tumors found that navoximod was well tolerated at doses up to 800 mg BID and was accompanied with decreased kynurenine levels in blood plasma [108].

Targeting tryptophan catabolism combined with other therapeutic strategies may improve the efficacy of cancer immunotherapy. This combination strategy has potential as an alternative for patients whose tumors do not respond to standard therapy [109]. Other therapeutic methods include, but are not limited to, checkpoint inhibitors, vaccination, and adoptive cell transfer therapy. Indoximod, an oral inhibitor of IDO1, plus docetaxel were well tolerated without an increase in toxicity and were active in a pretreated population of patients with metastatic solid tumors [110]. In a phase II trial, the combination therapy of indoximod with checkpoint inhibitors resulted in a 52% overall response rate in advanced melanoma patients [109]. Furthermore, navoximod combined with atezolizumab showed acceptable safety and tolerability for patients with advanced cancers; however, combination therapy did not result in a significant benefit [111].

### Other therapeutic targets

The spindle assembly checkpoint kinase TTK (Mps1), a key regulator of chromosome segregation, is a novel therapeutic target of small-molecule inhibitors. Treatment of a xenograft model of a CTNNB1-mutant cell line with the TTK inhibitor NTRC 0066-0 resulted in complete inhibition of tumor growth [112].

Small-molecule inhibitors or siRNA for targeting STAT3 signaling have also met with success in mice tumor models [113, 114]. Stattic, an inhibitor of STAT3, combined with metformin can inhibit tumor initiating cells in the brain by reducing STAT3-phosphorylation [115]. Moreover, combination therapy of recombinant vaccinia virus VG9 with Stattic was used to kill tumor cells by both oncolytic activity and inhibition of STAT3 phosphorylation; this combined strategy was superior to VG9 or Stattic alone [116].

Deregulation of the PI3K/Akt signaling pathway that leads to enhanced Akt activity is one of the most frequent changes in tumors. In a phase I trial (NCT01476657), duvelisib (an oral inhibitor of PI3K-δ/γ isoforms) demonstrated promising clinical activity and an acceptable safety profile in relapsed/refractory T cell lymphoma [117, 118]. The dual PI3K/mTOR inhibitor dactolisib also exhibited antitumor activity in vitro and in vivo [119] while omipalisib/GSK2126458 inhibited clonogenic growth in oncogenically transformed cells from neurocutaneous melanocytosis [120]. In addition, the Akt inhibitor Akti-1/2 was determined as an anticancer therapeutic drug [121].

When compared to parental cells, the cell invasion and migration abilities of OXA-R cells as well as the expression of metastasis-related markers decreased after treatment with the NF-κB inhibitor QNZ [122]. Moreover, the pH-sensitive co-delivery nanoparticle system of doxorubicin and pyrrolidinedithiocarbamate (PDTC, an inhibitor of NF-κB) showed promising potential for overcome multidrug resistance in breast cancer therapy [123]. SN50, a cell-permeable peptide inhibitor of NF-κB, results in decreased oncogenesis and induced differentiation of human glioma stem-like cells, suggesting that blocking the NF-κB signaling pathway is a potential therapeutic strategy for treating malignant brain tumors [124].

Finally, rapamycin was used for targeting TLR4, which triggered immune escape of tumor cells and inhibited the TLR4-activated NF-κB signaling pathway, uncovering a novel mechanism behind the antitumor effects of rapamycin [125].

## Conclusions

In this review, we discussed the mechanisms of how oncogenic signaling mediates tumor immune escape, which includes decreased effective immunocyte infiltration and function and increased levels of immunosuppressive cells in the tumor microenvironment (Fig. 3). Therefore, analyzing such tumor-intrinsic signaling pathways in patients with tumor progression/recurrence is critical as targeting these pathways is a promising strategy for cancer treatment (Fig. 3). The recent preclinical studies and clinical trials of targeting oncogenic signaling have shown encouraging results. We believe that oncogenic signaling-targeted therapies will be utilized for cancer patients in the future.

## Data Availability

The material supporting the conclusion of this review has been included within the article.

## Abbreviations

CAR-T: Chimeric antigen receptor-T
CCRK: Cell cycle-related kinase
CTLA-4: Cytotoxic T-lymphocyte-associated protein 4
DC: Dendritic cell
GBE1: Glycogen branching enzyme
IDO: Indoleamine-2,3-dioxygenase
MDSC: Myeloid-derived suppressor cell
NK: Natural killer
PD-1: Programmed cell death protein 1
RAGE: Advanced glycation end-products
TAM: Tumor-associated macrophage
TAN: Tumor-associated neutrophils
TDO: Tryptophan-2,3-dioxygenase 2
Treg: T regulatory cell
